# Stratified analysis of clinical pregnancy outcomes of sequential embryo transfer in frozen embryo transfer cycles based on different factors: a retrospective study

**DOI:** 10.1186/s12884-023-06111-5

**Published:** 2023-11-21

**Authors:** Jialing Li, Jing Ji, Hua Guo, Feimiao Wang, Yunxing Fu, Rong Hu

**Affiliations:** 1 Reproductive Medicine Center, General Hospital of Ningxia Medical University, Yinchuan, Ningxia 750004 China; 2 Institute of Medical Sciences, General Hospital of Ningxia Medical University, Yinchuan, Ningxia 750004 China; 3 Ningxia Medical University, Yinchuan, Ningxia 750004 China; 4 Department of Gynecology, General Hospital of Ningxia Medical University, Yinchuan, Ningxia 750004 China

**Keywords:** Sequential embryo transfer, Frozen embryo transfer cycle, Clinical pregnancy rate, Implantation rate, Repeated implantation failure

## Abstract

**Objective:**

To explore the effect of sequential embryo transfer (ET) on the pregnancy outcome of frozen-thawed embryo transfer (FET) cycle and the indications of sequential transfer.

**Methods:**

A total of 1440 FET cycles were enrolled in this retrospective study, of which 1080 patients received conventional ET and 360 patients received sequential ET. Further stratified analysis was performed according to the number of previous failed cycles, the number of embryos transferred and the stage of blastocyst (day 5 or 6, denoted D5 or D6) transferred. Comparison of pregnancy rates, implantation rate, miscarriage rate and multiple pregnancy rate among the groups of patients.

**Results:**

The clinical pregnancy rate and implantation rate of the sequential ET group were higher than those of the conventional ET group (P < 0.01); however, there was no statistical difference in multiple pregnancy rate and miscarriage rate (P > 0.05). In sequential transfer, the number of transferred embryos (2 or 3) and the stage of transferred blastocysts (D5 or D6) had no effect on clinical pregnancy rate, implantation rate, multiple pregnancy rate and miscarriage rate (P > 0.05). In patients with three or more previous failure cycles, the sequential ET group showed higher clinical pregnancy rate and implantation rate (P > 0.05).

**Conclusions:**

Compared with conventional ET in FET cycle, sequential ET strategy could significantly improve the clinical pregnancy rate and implantation rate. In sequential transfer, patients with three embryos transferred don’t have higher pregnancy rate and implantation rate. Besides, sequential transfer is more suitable for patients with repeated implantation failures (RIF), and increase the utilization rate of D6 blastocysts.

**Supplementary Information:**

The online version contains supplementary material available at 10.1186/s12884-023-06111-5.

## Introduction

Ovulation induction protocols, embryo culture systems and embryo freeze-thaw techniques in the laboratory have been continuously optimized during decades of development, resulting in the improved quantity and quality of embryos. However, the embryo implantation rate remains 25–40%, preventing in vitro fertilization-embryo transfer (IVF-ET) procedures from having an ideal outcome [1].

Embryo implantation is a critical initial stage of successful pregnancy involving multiple biological factors [2], the process of which requires embryos with high developmental potential, a receptive endometrium and effective dialogue between the two [3]. The required “cross-talk” leads to the apposition, attachment and invasion of embryos that is mandatory for successful implantation. Endometrial improvement is necessary to increase the success rate from IVF-ET. In murine experiments, it has been shown that embryos can induce improved endometrial receptivity [4]. In humans, Women with repeated IVF treatment failures had significantly higher rates of both implantation and pregnancy after sequential ET compared with a matched group of women who underwent transfer of day 3 embryos only [5]. However, at present, the effectiveness of sequential ET strategies remains controversial, and it is not clear whether it is applicable to all patients. As early as 1988, Abramovic and colleagues showed that a sequential ET protocol increased pregnancy rates [6]. However, studies by other investigators showed no significant differences in the pregnancy rate between conventional and sequential ET strategies [7, 8].

Repeated implantation failure (RIF) is determined when transferred embryos fail to implant in at least three repeated IVF attempts with 1–2 high-quality embryos in each cycle [3, 9]. In determining the time of embryo transfer, there is ultimately the need to hit the so-called window of implantation, a relatively short period of time when the endometrium is best suited to support embryo-endometrial interactions. Different timing for this window of implantation was found in at least 25% of patients experiencing RIF, and was based on transcriptomic modifications of the endometrium during the mid-luteal phase [10]. The best solution for patients with RIF is to implement a strategy that includes the optimal time for embryo transfer, as well as the appropriate developmental stage, to hit the optimal window of implantation (WOI). Studies focusing on sequential ET in patients with RIF demonstrated improved clinical pregnancy and implantation rates with sequential ET compared with regular day 2–3 ET [11–13]. However, there are also different views on the application of sequential transfer in patients with RIF [5, 14, 15]. There is still controversy about the influence of blastocyst quality and stage (day 5 or 6, denoted D5 or D6) on the clinical outcomes of blastocyst transfer [16–18]. Therefore, we also intended to explore whether blastocysts at different stages would affect clinical pregnancy outcomes in sequential transfer.

This retrospective study was aimed to stratify patients according to the number of previous failed cycles different number of embryos transferred (2 or 3) and different stages of blastocysts (D5 or D6) to further explore the clinical pregnancy outcomes of sequential transfer for FET, as well as the effectiveness, applicable population and safety of sequential transfer.

## Materials and methods

### Subjects and selection criteria

**Fig. 1:**
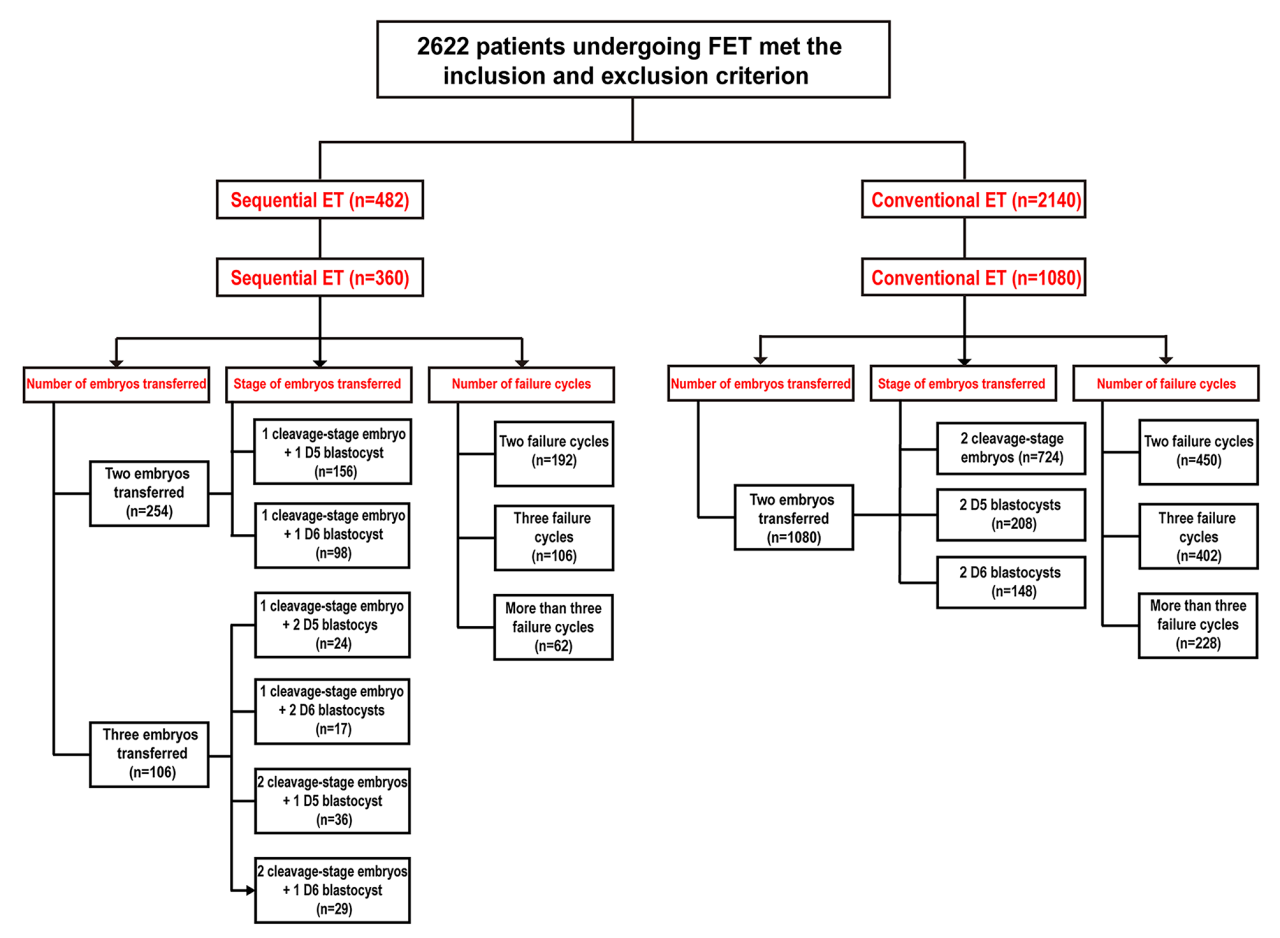
Flow chart showing the recruitment and grouping of study participants

This retrospective study finally recruited a total of 1440 subjects including conventional ET (n = 1080) and sequential ET (n = 360) by matching to cases in a 3:1 ratio base on years, BMI, AMH, bFSH, infertility duration and infertility factors. All FET cycles performed at the Reproductive Medicine Center of General Hospital of Ningxia Medical University from December 2017 to December 2021. The inclusion criterions were: patients undergoing FET using hormone placement therapy (HRT) to prepare the endometrium; patients with at least two failure cycles. The exclusion criterions were: abnormal chromosomal karyotype in the female or male; abnormal thrombosis screening or immune screening; sever intrauterine adhesions; uterine malformation; endometriosis; endometrial tuberculosis. The grouping of patients is shown in Fig. 1.

Of the 1080 patients in the conventional ET group, 724 patients received 2 cleavage-stage embryos, 208 patients received 2 D5-blastocysts, and 148 received 2 D6-blastocysts. In addition, 450 of the 1,080 had 2 previous failure cycles, 402 had 3 failure cycles, and 228 had more than 3 failure cycles.

In the sequential transfer group, 254 patients had 2 embryos transferred, of which 156 patients received 1 cleavage embryo + 1 D5-blastocyst and 98 received 1 cleavage embryo + 1 D6-blastocyst. 106 patients had 3 embryos transferred, of which 24 patients received 1 cleavage embryo + 2 D5-blastocysts, 17 patients received 1 cleavage embryo + 2 D6-blastocysts, 36 patients received 2 cleavage embryos + 1 D6-blastocyst, 29 patients received 2 cleavage embryos + 1 D6-blastocysts. In addition, 192 of the 360 had 2 previous failure cycles, 106 had 3 failure cycles, and 62 had more than 3 failure cycles.

This study was approved by the Reproductive Medicine Ethics Committee of General Hospital of Ningxia Medical University (2017 − 261). All patients were informed of the method of embryo transfer and signed the informed consent.

### Endometrial preparation and embryo transfer

On the 2nd to 3rd day of menstruation, the patients started to take 6–8 mg/d estradiol valerate tables (Proginao, Bayer, Germany), serum estradiol and progesterone levels were measured, and endometrium was monitored by vaginal ultrasonography. When the endometrium thickness reached 8 mm, progesterone (60 mg/d) was injected intramuscularly.

For the conventional ET group, cleavage-stage embryos were transferred on the 4th day of progesterone administration, or blastocysts were transferred on the 6th day of progesterone administration. For the sequential ET group, cleavage-stage embryos were transferred for the first time on the 4th day of progesterone administration, and D5 or D6 blastocysts were transferred again on the 6th day of progesterone administration.

In accord with the No. 176 document of the Ministry of Public Health of China *“Human Assisted Reproductive Technology Specifications”*, from the day of ET, patients were given dydrogesterone tablets (20 mg/d, Abbott Biologicals B.V., Duffton, The Netherlands). Luteal support was given with vaginal progesterone sustained-release gel (90 mg/d, Merck Serono, Snowrone, UK).

### Embryo assessment

Cleavage-stage embryo quality classification was mainly based on the size, shape and fragment ratio in blastomeres [19], as follows: grade I, blastomeres uniform in size, regular in shape, transparent, with a fragment ratio ≤ 10%; grade II, blastomeres slightly uneven in size or irregular in shape, with a few granules in the cytoplasm, and a fragment ratio of 10–20%; grade III, blastomeres obviously uneven in size or irregular in shape, with a number of granules in the cytoplasm, and a fragment ratio of 20–50%; grade IV, blastomeres severely uneven in size or irregular in shape, with cytoplasmic granulation, and a fragment ratio > 50%. High-quality embryos were defined as grade I and II embryos with two pronuclei on the first day after fertilization and developed to 6–10 blastomeres on the third day.

Blastocysts were divided into six stages on the basis of their degree of expansion and hatching status [20], as follows: stage 1, early blastocyst with a blastocoel less than half the volume of the embryo; stage 2, blastocyst with a blastocoel half or greater than half the volume of the embryo; stage 3, full blastocyst with a blastocoel completely filling the embryo; stage 4, expanded blastocyst with a blastocoel volume larger than that of the early embryo, with a thinning zona; stage 5, hatching blastocyst with the trophectoderm starting to herniate through the zona; and stage 6, hatched blastocyst that had completely escaped from the zona. Blastocysts were also graded according to the number and morphology of the inner cell mass (ICM) and the trophectoderm (TE). The development of the ICM was assessed as follows: A, tightly packed, many cells; B, loosely grouped, several cells; or C, very few cells. The TE was assessed as follows: A, many cells forming a cohesive epithelium; B, few cells forming a loose epithelium; or C, very few large cells.

High-quality embryos were defined as blastocysts at stage 3 and above, with ICM and TE reaching grade B and above on day 5, or at 4 and above, with the ICM and TE reaching grade B and above on day 6.

### Outcome measurements

The primary outcomes were clinical pregnancy, implantation, multiple pregnancy and miscarriage rates. Pregnancy testing was performed 14 days after ET. Ultrasound examination was performed at week 7 to assess fatal sac number and fetal heartbeat. Clinical pregnancy was defined as the presence of a fatal heart beat on ultrasound examination at 7 weeks of pregnancy. The implantation rate was defined as the number of gestational sacs detected by ultrasound divided by the total number of embryos/blastocysts transferred. Spontaneous miscarriage was defined as spontaneous loss of a clinical pregnancy before 22 completed weeks of gestational age. Multiple pregnancy was defined as two or more gestational sacs observed by ultrasound.

### Statistical analysis

The Statistical Package for Social Sciences 26.0 (SPSS, Chicago, IL, USA) was applied for statistical analysis. Quantitative data were expressed as mean ± standard deviation ($\bar x$ ± s), and the two independent-sample t -test was used for continuous variables. Qualitative data were expressed as percentages (%), and the Chi-squared test was used for comparison between the two groups. Multiple logistic regression model was used to adjust for confounding factors to observe the independent effects of sequential transplantation on pregnancy rate and multiple birth rate, Adjusted I and Adjusted II, were also presented in this study. Logistic regression analysis was performed using R 4.0.5. The significance level was set at a p value < 0.05.

## Results

### Analysis of baseline characteristics and pregnancy outcomes of patients in the sequential ET and conventional ET groups

As shown in Table 1, the average age, body mass index (BMI), infertility duration, anti-Mullerian hormone (AMH) level, base follicle stimulating hormone (bFSH) level and infertility factors in the sequential ET and conventional ET groups were not significantly different (*P* > 0.05). Besides, no statistically significant differences existed between the two groups with respect to the number of previous failed cycles, high-quality embryo rate and endometrial thickness on the day of ET (*P* > 0.05).

**Table 1:** Baseline characteristics and outcomes of patients in the sequential ET group and the conventional ET group ($\bar x$ ± s; %)

Indicators	Conventional ET (n = 1080)	Sequential ET (n = 360)	*P*-value
Age (yr)	31.73 ± 4.47	31.45 ± 4.32	0.388
BMI (kg/m^2^)	22.52 ± 3.53	22.62 ± 3.20	0.671
Infertility duration (yr)	3.73 ± 2.80	3.71 ± 2.67	0.938
AMH (ng/ml)	5.71 ± 4.81	5.56 ± 4.46	0.664
bFSH (mIU/ml)	6.68 ± 3.75	6.65 ± 2.21	0.890
Number of failed cycles	3.03 ± 1.69	3.00 ± 1.73	0.868
Endometrial thickness (mm)	10.92 ± 2.16	10.89 ± 1.92	0.801
High-quality embryos rate	43.0 (929/2160)	44.6 (368/826)	0.447
Primary infertility rate	53.6 (579/1080)	58.6 (211/360)	0.099
Infertility factors			
Male factors	19.7 (213/1080)	23.9 (86/360)	0.091
Tube and pelvic factors	42.5 (459/1080)	40.8 (147/360)	0.579
Ovulatory factors	6.1 (66/1080)	5.0 (18/360)	0.436
Couples’ factors	23.6 (255/1080)	20.0 (72/360)	0.157
Others	8.1 (87/1080)	10.3 (37/360)	0.193
Clinical pregnancy rate	23.6 (255/1080)	31.7 (114/360)	0.002**
Implantation rate	14.4 (310/2160)	17.9 (148/826)	0.010**
Multiple pregnancy rate	21.6 (55/255)	22.8 (26/114)	0.791
Miscarriage rate	11.8 (30/255)	13.2 (15/114)	0.706

Note: BMI = Body mass index; AMH = anti-Mullerian hormone; bFSH = base follicle stimulating hormone. **P < 0.01

Compared with the conventional ET group, the clinical pregnancy and implantation rates were significantly higher in the sequential ET group (23.6% vs. 31.7%, *P* = 0.002; 14.4% vs. 17.9%, *P* = 0.01). The multiple pregnancy and miscarriage rates were not significantly different between the two groups (21.6% vs. 22.8%, 11.8% vs. 13.2%, respectively, *P >* 0.05).

Several sets of regression models were listed according to the STROBE statement, including the crude model, adjusted model 1 and adjusted model 2. No confounding factors were adjusted in the crude model; Adjusted model 1 corrected age, BMI, infertility years and endometrial thickness on the day of ET, and adjusting model 2 corrected AMH, bFSH, number of failed cycles and number of embryos transferred on the basis of adjusting model 1. After adjusting for confounders, the clinical pregnancy rate of patients in the sequential ET increased by 42% (OR = 1.42, 95%CI: 1.16, 1.97) (Supplementary Table 1). Next, we made a multivariable regression model for the multiple pregnancy rate of the two groups of patients, also including the crude model, adjusted model 1 and adjusted model 2. As shown in Supplementary Tables 2, Sequential ET did not increase the risk of multiple pregnancy rate of the patients (OR = 1.15, 95%CI: 0.78, 1.81).

#### Analysis of baseline characteristics and pregnancy outcomes of patients in two groups with two embryos transferred

In order to eliminate the influence of embryo number on pregnancy outcome, patients in the sequential ET group with two embryos transferred were screened and compared with patients in the conventional ET group.

As shown in Table 2, for patients with two embryos transferred, the average age, BMI, infertility duration, AMH level, bFSH level, infertility factors, number of previous failed cycles, high-quality embryo rate and endometrial thickness on the day of ET were not significantly different between the conventional and sequential ET groups (*P* > 0.05). However, compared with the conventional ET group, the sequential ET group had higher clinical pregnancy and implantation rates (23.6% vs. 30.3%, P = 0.026; 14.4% vs. 18.3%, *P* = 0.025). Multiple pregnancy and miscarriage rates were not significantly different between the two groups (21.6% vs. 23.4%, 11.8% vs. 15.6%, respectively, *P >* 0.05). After controlling for the number of embryos transferred, sequential transplantation still showed an advantage.

**Table 2:** Baseline characteristics and pregnancy outcomes of patients with two embryos transferred in two groups ($\bar x$ ± s; %)

Indicators	Two embryos transferred		
	Conventional ET (1080)	Sequential ET (n = 254)	*P*-value
Age (yr)	31.73 ± 4.47	31.41 ± 4.18	0.357
BMI (kg/m^2^)	22.52 ± 3.53	22.61 ± 3.19	0.682
Infertility duration (yr)	3.73 ± 2.80	3.57 ± 2.71	0.637
AMH (ng/ml)	5.71 ± 4.81	5.70 ± 4.76	0.978
bFSH (mIU/ml)	6.68 ± 3.75	6.87 ± 2.19	0.363
Number of failed cycles	3.03 ± 1.69	2.83 ± 1.57	0.167
Endometrial thickness (mm)	10.92 ± 2.16	10.88 ± 1.98	0.751
High-quality embryos rate	43.0 (929/2160)	44.9 (228/508)	0.443
Primary infertility rate	53.6 (579/1080)	59.8 (152/254)	0.073
Infertility factors			
Male factors	19.7 (213/1080)	24.0 (61/254)	0.128
Tube and pelvic factors	42.5 (459/1080)	40.2 (102/254)	0.474
Ovulatory factors	6.1 (66/1080)	5.1 (13/254)	0.546
Couples’ factors	23.6 (255/1080)	20.9 (53/254)	0.350
Others	8.1 (87/1080)	9.8 (25/254)	0.355
Clinical pregnancy rate	23.6 (255/1080)	30.3 (77/254)	0.026*
Implantation rate	14.4 (310/2160)	18.3 (93/508)	0.025*
Multiple pregnancy rate	21.6 (55/255)	23.4 (16/77)	0.737
Miscarriage rate	11.8 (30/255)	15.6 (12/77)	0.377

Note: BMI = Body mass index; AMH = anti-Mullerian hormone; bFSH = base follicle stimulating hormone. *P < 0.05

### Stratified analysis of baseline characteristics and pregnancy outcomes of patients in the sequential ET group with different numbers of embryos transferred

As shown in Table 3, in the sequential ET group, number of previous failed cycles for patients with two embryos transferred was lower than that of patients with three embryos transferred. In addition, there was no statistical difference in other general conditions between two versus three embryos transferred (*P* > 0.05). Similarly, there were no statistical differences in the clinical pregnancy, implantation, multiple pregnancy and miscarriage rates (*P* > 0.05).

**Table 3:** Baseline characteristics and pregnancy outcomes of patients with different numbers of embryos transferred in the sequential ET group ($\bar x$ ± s; %)

Indicators	Two embryos transferred (n = 254)	Three embryos transferred (n = 106)	*P*-value
Age (yr)	31.41 ± 4.18	31.56 ± 4.67	0.763
BMI (kg/m^2^)	22.61 ± 3.19	22.65 ± 3.24	0.910
Infertility duration (yr)	3.57 ± 2.71	4.07 ± 2.56	0.107
AMH (ng/ml)	5.70 ± 4.76	5.23 ± 3.66	0.357
bFSH (mIU/ml)	6.87 ± 2.19	6.11 ± 2.19	0.053
Number of failed cycles	2.83 ± 1.57	3.41 ± 2.02	0.009**
Endometrial thickness (mm)	10.88 ± 1.98	10.89 ± 1.79	0.969
High-quality embryos rate	44.9 (228/508)	44.0 (140/318)	0.810
Primary infertility rate	59.8 (152/254)	55.7 (59/106)	0.463
Infertility factors			
Male factors	24.0 (61/254)	23.6 (25/106)	0.930
Tube and pelvic factors	40.2 (102/254)	42.5 (45/106)	0.686
Ovulatory factors	5.1 (13/254)	4.7 (5/106)	0.874
Couples’ factors	20.9 (53/254)	17.9 (19/106)	0.525
Others	9.8 (25/254)	11.3 (12/106)	0.674
Clinical pregnancy rate	30.3 (77/254)	34.9 (37/106)	0.393
Implantation rate	18.3 (93/508)	17.3 (55/318)	0.712
Multiple pregnancy rate	20.8 (16/77)	27.0 (10/37)	0.457
Miscarriage rate	15.6 (12/77)	8.1 (3/37)	0.269

Note: BMI = Body mass index; AMH = anti-Mullerian hormone; bFSH = base follicle stimulating hormone. **P < 0.01

### Stratified analysis of patients in the sequential ET and conventional ET groups based on the stage of blastocyst development

#### Analysis of baseline characteristics and pregnancy outcomes of patients with D5/D6 blastocyst transferred

As shown in Table 4, the average age, BMI, infertility duration, AMH level, FSH level, infertility factors, number of previous failed cycles, high-quality embryo rate and endometrial thickness in the sequential transfer of the D5 blastocyst group and conventional transfer of the D5 blastocyst group were not significantly different (*P* > 0.05). Compared with the conventional ET group, there were significant differences between the clinical pregnancy rate and implantation rate in the sequential ET(24.5% vs. 33.8%, *P* = 0.036; 17.0% vs. 19.6%, *P* = 0.038). No significant differences between the multiple pregnancy and miscarriage rates in the sequential transfer of the D5 blastocyst group (17.6% vs. 24.7% and 11.8% vs. 12.3%, respectively, *P >* 0.05).

**Table 4:** Baseline characteristics and pregnancy outcomes of patients with different stages of blastocysts transferred in two groups ($\bar x$ ± s; %)

Indicators	D5 blastocyst transferred			D6 blastocyst transferred		
	Conventional ET (n = 208)	Sequential ET (n = 216)	*P*-value	Conventional ET (n = 148)	Sequential ET (n = 144)	*P*-value
Age (yr)	31.78 ± 4.58	31.76 ± 4.52	0.968	31.40 ± 3.95	31.69 ± 4.41	0.596
BMI (kg/m^2^)	22.74 ± 3.34	22.77 ± 3.75	0.935	22.48 ± 3.10	22.34 ± 3.19	0.737
Infertility duration (yr)	3.74 ± 2.72	3.78 ± 2.85	0.877	3.44 ± 2.59	3.69 ± 2.70	0.452
AMH (ng/ml)	5.38 ± 4.64	5.70 ± 4.90	0.501	5.75 ± 4.35	5.76 ± 4.78	0.984
bFSH (mIU/ml)	6.82 ± 2.45	6.81 ± 4.13	0.977	6.55 ± 2.10	6.52 ± 3.00	0.926
Number of failed cycles	2.97 ± 1.76	3.16 ± 1.64	0.265	3.28 ± 1.53	3.33 ± 1.77	0.112
Endometrial thickness (mm)	10.94 ± 1.92	10.74 ± 2.08	0.319	10.92 ± 2.06	11.06 ± 2.26	0.718
High-quality embryos rate	41.3 (172/416)	45.1 (223/494)	0.250	37.7 (86/228)	42.5 (141/332)	0.261
Primary infertility rate	51.9 (108/208)	59.7 (129/216)	0.106	58.8 (87/148)	56.9 (82/144)	0.750
Infertility factors						
Male factors	22.1 (46/208)	24.5 (53/216)	0.556	27.0 (40/148)	22.9 (33/144)	0.417
Tube and pelvic factors	42.3 (88/208)	43.1 (93/216)	0.876	41.9 (62/148)	37.5 (54/144)	0.443
Ovulatory factors	3.8 (8/208)	5.6 (12/216)	0.407	3.4 (5/148)	4.2 (6/144)	0.724
Couples’ factors	18.3 (38/208)	19.0 (41/216)	0.851	20.3 (30/148)	24.3 (35/144)	0.407
Others	13.5 (28/208)	7.9 (17/216)	0.062	7.4 (11/148)	11.1 (16/144)	0.278
Clinical pregnancy rate	24.5 (51/208)	33.8 (73/216)	0.036*	17.6 (26/148)	28.5 (41/144)	0.027*
Implantation rate	17.0 (60/416)	19.6 (97/494)	0.038*	10.1 (30/296)	15.4 (51/332)	0.051
Multiple pregnancy rate	17.6 (9/51)	24.7 (18/73)	0.352	15.4 (4/26)	19.5 (8/41)	0.668
Miscarriage rate	11.8 (6/51)	12.3 (9/73)	0.924	15.4 (4/26)	14.6 (6/41)	0.933

Note: BMI = Body mass index; AMH = anti-Mullerian hormone; bFSH = base follicle stimulating hormone. *P < 0.05

The clinical pregnancy rate of the two groups of patients with D6 blastocysts was significant differences between two groups (17.6% vs. 28.5%, *P* = 0.027). Even if there was no statistical difference, possibly due to insufficient sample size, it could still be seen from the results that the implantation rate of patients in the sequential ET group was higher than that in the conventional ET group (10.1% vs. 15.4%, *P* = 0.051).

#### Analysis of baseline characteristics and pregnancy outcomes of patients in the sequential ET group with D5/D6 blastocyst transferred 

**Table 5:** Baseline characteristics and pregnancy outcomes of patients with different stages of blastocyst transferred in the sequential ET group ($\bar x$ ± s; %)

Indicators	D5 blastocyst transferred (n = 216)	D6 blastocyst transferred (n = 144)	*P*-value
Age (yr)	31.76 ± 4.52	31.69 ± 4.41	0.874
BMI (kg/m^2^)	22.77 ± 3.75	22.34 ± 3.19	0.261
Infertility duration (yr)	3.78 ± 2.85	3.69 ± 2.70	0.770
AMH (ng/ml)	5.70 ± 4.90	5.76 ± 4.78	0.901
bFSH (mIU/ml)	6.81 ± 4.13	6.52 ± 3.00	0.463
Number of failed cycles	3.16 ± 1.64	3.33 ± 1.77	0.348
Number of embryos transferred	2.27 ± 0.45	2.31 ± 0.47	0.397
Endometrial thickness (mm)	10.74 ± 2.08	11.06 ± 2.26	0.139
High-quality cleavage-stage embryo rate	43.3 (101/233)	40.4 (76/188)	0.546
Primary infertility rate	59.7 (129/216)	56.9 (82/144)	0.600
Infertility factors			
Male factors	24.5 (53/216)	22.9 (33/144)	0.724
Tube and pelvic factors	43.1 (93/216)	37.5 (54/144)	0.293
Ovulatory factors	5.6 (12/216)	4.2 (6/144)	0.554
Couples’ factors	19.0 (41/216)	24.3 (35/144)	0.225
Others	7.9 (17/216)	11.1 (16/144)	0.297
Clinical pregnancy rate	33.8 (73/216)	28.5 (41/144)	0.287
Implantation rate	19.6 (97/494)	15.4 (51/332)	0.116
Multiple pregnancy rate	24.7 (18/73)	19.5 (8/41)	0.530
Miscarriage rate	12.3 (9/73)	14.6 (6/41)	0.727

Note: BMI = Body mass index; AMH = anti-Mullerian hormone; bFSH = base follicle stimulating hormone

As shown in Table 5, in the sequential ET group, the average age, infertility duration, AMH level, FSH level, number of previous failed cycles, endometrial thickness and high-quality cleavage-stage embryo rate were equivalent in patients with D5 or D6 blastocyst transfer (*P* > 0.05). Similarly, the clinical pregnancy, implantation, multiple pregnancy and miscarriage rates showed no significant differences between these two subgroups (*P* > 0.05).

### Stratified analysis of patients in the sequential ET and conventional ET groups based on the number of previous failure cycles

As shown in Table 6, the average age, BMI, infertility duration, AMH, bFSH, endometrial thickness, number of embryos transferred, high-quality embryo rate and infertility factors were not significantly different in patients in each group.

**Table 6:** Baseline characteristics and pregnancy outcomes of patients with different failure cycles in two groups ($\bar x$ ± s; %)

Indicators	with two failure cycles			with three failure cycles			with more than three failure cycles		
	Conventional ET (n = 450)	Sequential ET (n = 192)	*P*-value	Conventional ET (n = 402)	Sequential ET (n = 106)	*P*-value	Conventional ET (n = 228)	Sequential ET (n = 62)	*P*-value
Age (yr)	31.17 ± 4.11	31.36 ± 4.43	0.672	32.18 ± 4.68	31.20 ± 4.04	0.088	32.07 ± 4.72	32.16 ± 4.46	0.920
BMI (kg/m^2^)	22.65 ± 3.41	22.54 ± 3.15	0.755	22.42 ± 3.8	22.66 ± 3.3	0.616	23.41 ± 3.30	22.82 ± 3.21	0.468
Infertility duration (yr)	3.64 ± 2.49	4.20 ± 2.97	0.061	3.88 ± 2.96	3.25 ± 3.27	0.071	3.66 ± 3.11	3.04 ± 2.01	0.160
AMH (ng/ml)	6.50 ± 5.18	5.66 ± 4.20	0.098	5.38 ± 4.54	5.69 ± 5.27	0.624	4.73 ± 4.30	5.03 ± 3.75	0.671
bFSH (mIU/ml)	6.36 ± 2.83	6.44 ± 2.71	0.798	6.54 ± 2.49	7.03 ± 2.60	0.135	7.03 ± 4.91	6.29 ± 2.08	0.234
Endometrial thickness (mm)	10.69 ± 2.36	10.94 ± 1.95	0.293	11.04 ± 1.85	10.74 ± 1.84	0.205	11.17 ± 2.23	10.97 ± 1.99	0.586
High-quality enbryo rate	48.1 (433/900)	45.3 (193/426)	0.339	39.9 (321/804)	42.2 (105/249)	0.529	38.4 (175/456)	46.4 (70/151)	0.083
Primary infertlity rate	58.7 (264/450)	64.1 (123/192)	0.201	51.5 (207/402)	60.4 (64/106)	0.103	47.4 (108/228)	38.7 (24/62)	0.225
Infertility factors									
Male factors	20.0 (90/450)	25.5 (49/192)	0.120	19.4 (78/402)	19.8 (21/106)	0.925	19.7 (45/228)	25.8 (16/62)	0.298
Tube and pelvic factors	46.0 (207/450)	42.7 (82/192)	0.443	44.0 (177/402)	44.3 (47/106)	0.954	32.9 (75/228)	29.0 (18/62)	0.563
Ovulatory factors	6.7 (30/450)	6.3 (12/192)	0.845	6.0 (24/402)	3.8 (4/106)	0.378	5.3 (12/228)	3.2 (2/62)	0.507
Bilateral factors	21.3 (96/450)	17.2 (33/192)	0.230	23.1 (93/402)	23.6 (25/106)	0.922	28.9 (66/228)	22.6 (14/62)	0.320
Others	6.0 (27/450)	8.3 (16/192)	0.279	7.5 (30/402)	8.5 (9/106)	0.724	13.2 (30/228)	19.4 (12/62)	0.219
Clinical pregnancy rate	33.3 (150/450)	35.4 (68/192)	0.610	19.4 (78/402)	31.1 (32/106)	0.020*	11.8 (27/228)	22.6 (14/62)	0.031*
Implantation rate	20.1 (181/900)	20.2 (86/426)	0.974	11.9 (96/804)	18.1 (45/249)	0.013*	7.2 (33/456)	12.6 (19/151)	0.040*
Multiple pregnancy rate	20.7 (31/150)	22.1 (15/68)	0.815	23.1 (18/78)	25.0 (8/32)	0.829	22.2 (6/27)	21.4 (3/14)	0.954
Miscarriage rate	16.0 (24/150)	16.2 (11/68)	0.974	7.7 (6/78)	9.4 (3/32)	0.770	-	5.6 (1/18)	-

Note: BMI = Body mass index; AMH = anti-Mullerian hormone; bFSH = base follicle stimulating hormone. *P < 0.05

In the patients with two failure cycles, there were no significant differences in the clinical pregnancy rate, implantation rate, multiple pregnancy rate and miscarriage rate between the sequential ET group and the conventional ET group (*P* > 0.05). But sequential ET showed clear advantages in the clinical pregnancy rate and implantation rate in once patients had three or more than three failure cycles (*P*<0.05).

## Discussion

The clinical pregnancy rate of IVF is usually 40–50%, and can be high as 60% in patients receiving IVF treatment for the first time [21]. Studies have shown that two-thirds of ET failures are due to lack of endometrial receptivity, and one-third are due to poor embryo quality [22]. Therefore, in advanced reproductive centers, assuming a good embryo culture environment, improving endometrial receptivity is essential to increase the success rate of IVF-ET.

In this study, clinical pregnancy and implantation rates were higher in the sequential ET group than in the conventional ET group. It is possible that the first transferred cleavage-stage embryos induced an increase in endometrial receptivity, thus creating a better endometrium microenvironment for the second ET. Mercader et al. found that co-culture of early-stage embryos with endometrial epithelial cells yielded blastocyst formation rates of 50.8–58.2% with suitable implantation, which increased the pregnancy rate of IVF [23]. The cleavage stage ET for the first time increases the chance of synchronizing with endometrial development, providing a better endometrial environment for re-transfer of blastocyst. Also, insertion of the catheter during the first transfer may induce mechanical stimulation of the endometrium, resulting the endometrium to release inflammatory factors that promote embryo adhesion during the second transfer [24, 25]. In addition, WOI may last a few hours or a few days. some investigators reported that the transfer of two embryos at different periods can increase the chance of embryos hitting the optimal endometrial WOI, thus increasing the success rate of IVF [12, 13]. The current study found that the multiple birth rate was not statistically different in the sequential ET group compared with the conventional ET group, consistent with the results of Goto et al., indicating that sequential ET may increase implantation and pregnancy rates but not increase the risk of multiple births [26].

Studies have shown that compared with fresh embryo transfer ET, the weight of single newborns id higher in freeze-thaw ET cycles, and the incidence of small for gestational age (SGA) and fetal growth restriction (FGR) is lower [27, 28]. And IVF/ICSI conceptions with thawed as opposed to fresh blastocyst transfer present a lower mean uterine pulsatility index [29, 30]. Patients undergoing freeze-thaw ET have an environment in utero closer to that of natural pregnancy [31]. To further explore the suitable population for sequential ET in the frozen embryo cycle, the present study compared the number of previous failed cycles for the patients. This analysis showed that the clinical pregnancy rate and implantation rate were higher in the sequential ET group than in the conventional ET group for patients who had three failed cycles, and the same outcomes shown in patients who had more than three failed cycles in the past. In patients only having two failed cycles, sequential transfer and conventional transfer groups had similar pregnancy outcomes. These findings indicate that the sequential ET strategy may not be suitable for all patients and may be more applicable for patients with RIF. For patients undergoing their first or second transfer, sequential ET provided no obvious advantage, so sequential ET may waste embryos without improving clinical outcomes. Sequential ET for frozen embryo cycles for patients with RIF provides an effective treatment method and can increase the implantation and clinical pregnancy rates without increasing the risk of multiple pregnancy, infection, and low birth weight and ectopic pregnancy rates [14, 15, 32].

Studies have shown that the transfer of blastocysts at different days of development will affect the clinical outcomes of IVF [33, 34]. A meta-analysis indicated that the clinical pregnancy rate was higher from D5 blastocyst transfer than that from D6 blastocyst transfer during fresh embryo and FET cycles [35]. In FET, Muthukumar et al. found that the clinical pregnancy and implantation rates were higher from D5 blastocyst transfers than from D6 blastocyst transfers [36]. Dessolle et al. reported that healthy term birth rate was significantly higher from D5 blastocyst transfers than that from D6 blastocyst transfers, and suggested avoiding D6 blastocyst transfers because blastocyst stage was an important factor related to pregnancy outcomes [37]. There is also research suggesting that the aneuploidy rate of D5 blastocysts is lower than that of D6 blastocysts [38]. In terms of the dynamics of embryonic development, past work showed that D6 blastocysts develop more slowly and have lower developmental potential than D5 blastocysts [39]. The results of this study suggested that compared with the patients with conventional transfer of the D5/D6 blastocyst group, the clinical pregnancy rate and implantation rate were significant higher in patients with sequential ET. However, there was no difference in pregnancy outcomes from D5 versus D6 blastocysts in the sequential ET group based on the similar high-quality cleavage-stage embryo rate, indicating that sequential transfer may improve the utilization rate of D6 blastocysts to achieve the same results as D5 blastocysts. This proposal requires further stratified analysis of cleavage-stage embryo quality and validation in a large sample prospective study.

Besides, the present study compared the pregnancy outcomes of the two groups of patients with different numbers of transferred embryos to further examine the impact of the number of embryos transferred upon IVF outcomes. This study found that the clinical pregnancy and implantation rates were higher in the sequential ET group than in the conventional ET group among the patients receiving two embryos, and there was no statistical difference in the multiple birth and miscarriage rates. These findings indicate that the sequential transfer of two embryos can effectively improve the success rate of patients undergoing frozen embryo cycles and will not increase the risk of multiple birth and miscarriage. The current findings also showed that the number of transferred embryos in the sequential ET group (one cleavage-stage embryo + one blastocyst vs. one cleavage-stage embryo + two blastocysts / two cleavage-stage embryos + one blastocyst) had no effect on the clinical outcomes of sequential transfer. Therefore, when the sequential transfer strategy is adopted, only one cleavage-stage embryo and one blastocyst should be transferred, to increase the success rate of the frozen embryo cycle and avoid waste of embryos. It is recommended that each cleavage-stage embryo or blastocyst should be frozen separately for patients with RIF, providing a convenient process for subsequent serial transfer of a cleavage-stage embryo and a blastocyst. However, the number of previous failed cycles for patients with two embryos transferred was lower than that of patients with three embryos transferred in the sequential ET group, and the difference was statistically significant. Therefore, this finding should be interpreted with caution, and further large-sample studies are needed.

In summary, sequential ET can improve the success rate of frozen ET, but the number of transferred embryos should be controlled. It is recommended that a double ET strategy of one cleavage-stage and one blastocyst-stage embryo in sequential transfer be used to achieve beneficial results. While achieving a similar pregnancy outcome, embryo waste is reduced. For sequential ET, D5 or D6 blastocysts had no significant effect on the pregnancy outcome. Also, sequential ET is more suitable for patients with previous unsuccessful IVF-ET cycles. In patients with more than two cycle of previous failure, sequential ET can improve their pregnancy outcomes.

A limitation of this study is that it did not investigate whether sequential ET increased the pregnancy rate from frozen embryos while also increasing the financial burden of patients. Therefore, it is necessary to further compare the cost-effectiveness of these two transfer methods to achieve pregnancy for patients. In addition, we did not evaluate postnatal outcomes of the fetus, such as the rates of premature birth and low birth weight infants. Most importantly, the inclusion criteria for patientd in this study was that patients must have enough failed cycles and frozen embryos, which was relatively strict, so the final clinical cases were limited. These preliminary findings need to be further investigated in large-scale randomized trials to better guide potential clinical applications.

### Electronic supplementary material

Below is the link to the electronic supplementary material.

Supplementary Material 1

## Data Availability

The datasets used and/or analyzed during the present study are available from the corresponding author on reasonable request.

## Abbreviations

FET: Frozen-thawed Embryo Transfer
ET: Embryo Transfer
D5: Day5
D6: Day6
RIF: Repeated Implantation Failures
IVF-ET: In Vitro Fertilization-Embryo Transfer
WOI: window of implantation
HRT: Hormone Placement Therapy
ICM: Inner Cell Mass
TE: Trophectoderm
BMI: Body Mass Index
AMH: Anti-Mullerian Hormone
bFSH: base Follicle Stimulating Hormone
SGA: small for gestational age
FGR: fetal growth restriction
