# Supplementary material for: Stratified analysis of clinical pregnancy outcomes of sequential embryo transfer in frozen embryo transfer cycles based on different factors: a retrospective study
Source: BMC Pregnancy Childbirth. 2023 Nov 21;23:806. doi: 10.1186/s12884-023-06111-5 (PMC10664651; doi:10.1186/s12884-023-06111-5)
Supplement: Supplementary file 1 — Supplementary Material 1 [file 12884_2023_6111_MOESM1_ESM.docx]

Supplementary Table 1 Multivariate regression analysis of pregnancy rate in two groups.

| Model | **Method** | | *P*-value |
| --- | --- | --- | --- |
|  | Conventional ET | Sequential ET |  |
| Case/Total | 255/1080 | 114/360 |  |
| Crude model | 1.00 (ref.) | 1.33 (1.05, 1.87) | 0.003 |
| Adjusted model 1 | 1.00 (ref.) | 1.39 (1.11, 1.92) | 0.003 |
| Adjusted model 2 | 1.00 (ref.) | 1.42 (1.16, 1.97) | 0.002 |

Supplementary Table 2. Multivariate regression analysis of multiple pregnancy rate in two groups.

| Model | **Method** | | *P*-value |
| --- | --- | --- | --- |
|  | Conventional ET | Sequential ET |  |
| Case/Total | 55/255 | 26/114 |  |
| Crude model | 1.00 (ref.) | 1.07 (0.63, 1.62) | 0.791 |
| Adjusted model 1 | 1.00 (ref.) | 1.10 (0.71, 1.71) | 0.733 |
| Adjusted model 2 | 1.00 (ref.) | 1.15 (0.78, 1.81) | 0.582 |
